# Distinct patterns of APP processing in the CNS in autosomal-dominant and sporadic Alzheimer disease

**DOI:** 10.1007/s00401-012-1062-9

**Published:** 2012-12-06

**Authors:** Marta Pera, Daniel Alcolea, Raquel Sánchez-Valle, Cristina Guardia-Laguarta, Martí Colom-Cadena, Nahuai Badiola, Marc Suárez-Calvet, Albert Lladó, Alvaro A. Barrera-Ocampo, Diego Sepulveda-Falla, Rafael Blesa, José L. Molinuevo, Jordi Clarimón, Isidre Ferrer, Ellen Gelpi, Alberto Lleó

**Affiliations:** 1 Department of Neurology, Inst. Investigacions Biomediques, Hospital de Sant Pau, Universitat Autònoma de Barcelona, Sant Antoni Mª Claret, 167, 08025 Barcelona, Spain; 2 Centro de Investigación Biomédica en Red en enfermedades Neurodegenerativas, CIBERNED, Madrid, Spain; 3 Alzheimer’s Disease and other Cognitive Disorders Unit, Department of Neurology, Hospital Clínic, Barcelona, Spain; 4 Institute of Neuropathology, University Medical Center Hamburg-Eppendorf, Hamburg, Germany; 5 Grupo de Neurociencias de Antioquia, Faculty of Medicine, University of Antioquia, Medellín, Colombia; 6 Institut de Neuropatología, Servei Anatomia Patológica, IDIBELL, Hospital Universitari de Bellvitge, University of Barcelona, Barcelona, Spain; 7 Neurological Tissue Bank, Biobanc-Hospital Clínic-IDIBAPS, Barcelona, Spain

**Keywords:** Amyloid precursor protein, Autosomal-dominant Alzheimer disease, *β*-Site APP-cleaving enzyme, Presenilin, *β*-Amyloid

## Abstract

Autosomal-dominant Alzheimer disease (ADAD) is a genetic disorder caused by mutations in Amyloid Precursor Protein (*APP*) or Presenilin (*PSEN*) genes. Studies from families with ADAD have been critical to support the amyloid cascade hypothesis of Alzheimer disease (AD), the basis for the current development of amyloid-based disease-modifying therapies in sporadic AD (SAD). However, whether the pathological changes in APP processing in the CNS in ADAD are similar to those observed in SAD remains unclear. In this study, we measured *β*-site APP-cleaving enzyme (BACE) protein levels and activity, APP and APP C-terminal fragments in brain samples from subjects with ADAD carrying *APP* or *PSEN1* mutations (*n* = 18), patients with SAD (*n* = 27) and age-matched controls (*n* = 22). We also measured sAPP*β* and BACE protein levels, as well as BACE activity, in CSF from individuals carrying *PSEN1* mutations (10 mutation carriers and 7 non-carrier controls), patients with SAD (*n* = 32) and age-matched controls (*n* = 11). We found that in the brain, the pattern in ADAD was characterized by an increase in APP *β*-C-terminal fragment (*β*-CTF) levels despite no changes in BACE protein levels or activity. In contrast, the pattern in SAD in the brain was mainly characterized by an increase in BACE levels and activity, with less APP *β*-CTF accumulation than ADAD. In the CSF, no differences were found between groups in BACE activity or expression or sAPP*β* levels. Taken together, these data suggest that the physiopathological events underlying the chronic A*β* production/clearance imbalance in SAD and ADAD are different. These differences should be considered in the design of intervention trials in AD.

**Electronic supplementary material:**

The online version of this article (doi:10.1007/s00401-012-1062-9) contains supplementary material, which is available to authorized users.

## Introduction

Autosomal-dominant Alzheimer disease (ADAD) is a genetic disorder that accounts for less than 1 % of all AD cases [6]. It is genetically heterogeneous and has been associated with mutations in the amyloid precursor protein (*APP*) gene or in the two presenilin genes (presenilin-1 and -2 or *PSEN1* and *PSEN2*) [6].

Studies in ADAD have been critical to support the amyloid cascade hypothesis, which states that the sequence of pathogenic events leading to AD is primarily initiated by accumulation of *β*-amyloid (A*β*) [24]. The knowledge derived from these studies has been instrumental in guiding the development of the amyloid-based disease-modifying drugs currently being tested in sporadic Alzheimer disease (SAD).

A*β* peptide is the major protein component of amyloid plaques observed in the brain of patients with ADAD and SAD and it is produced via sequential cleavage of APP by two proteases, *β*- and *γ*-secretases [37]. The prevailing view about the cause of brain A*β* deposition in ADAD is that *APP* and *PSEN* mutations lead to a chronic increase in the absolute or relative production of the fibrillogenic 42-aminoacid-long form of A*β* (A*β*42) that, over time, leads to formation of brain oligomeric A*β*, deposition of fibrillar A*β* and eventually neurodegeneration [6]. The causes of A*β* accumulation in SAD are far more complex. A predominant view claims that brain A*β* deposition in SAD results from the complex interaction of genetic and environmental factors that end up in a chronic imbalance between A*β* production and clearance. Different mechanisms have been proposed to explain this chronic imbalance in SAD, such as increased [19, 26, 27, 34, 58], altered production [25] or reduced clearance of A*β* [38]. The investigation to elucidate the molecular mechanisms of AD has been complicated by the fact that many studies about the pathogenesis of AD rely on transgenic mouse models that overexpress ADAD-associated mutations. The results of these investigations are often extrapolated to all forms of AD, irrespective of the underlying causes. Elucidating the differences and commonalities between ADAD and SAD in the human CNS is an important topic as the first intervention trials in preclinical and presymptomatic AD are imminent. Although some previous studies have focused on the differences in A*β* isoforms between ADAD and SAD in the CNS [44–46, 49], other aspects of APP processing remain poorly investigated. In this study, we focused on BACE protein and activity, and their related cleavage products in a large a collection of well-characterized brain and CSF samples from subjects with ADAD carrying *APP* or *PSEN1* mutations, patients with SAD and age-matched controls.

## Materials and methods

### Human brain samples

All individuals or relatives had given their written informed consent for research and the study was approved by the local ethical standards committee on human experimentation. Human brain samples were obtained from the Institut de Neuropatologia, Hospital Universitari de Bellvitge, and the Neurological Tissue Bank of the Biobanc-Hospital Clinic-IDIBAPS. We included samples from 10 patients with ADAD (2 with an *APP* mutation and 8 with *PSEN1* mutations, mean age 55 ± 8.7 years, Table 1) [2, 23, 35, 36], 19 patients with SAD (mean age 78 ± 8.0 years, Braak neurofibrillary stage = V–VI, Thal phase of A*β* = 5), and 22 healthy controls (Braak neurofibrillary stage = 0; 12 young controls and 10 elderly controls, mean age 48.7 ± 13.2 and 75.1 ± 6.5 years, respectively). The mean postmortem interval (PMI) was 7.4 ± 4.8 h. As a confirmation group we included 8 additional cases with the E280A *PSEN1* mutation (mean age 54.5 ± 4.9 years) and 8 age-matched cases with SAD from the University of Antioquia Brain Bank (Table 1) [52, 53]. For biochemical analyses we used frozen blocks from the frontal association cortex, known to have high density of amyloid plaques [3, 34]. For immunohistochemical analyses paraffin-embedded samples from several brain regions were used (see below).

**Table 1:** Clinical and neuropathological data of ADAD patients from whom brain material was analyzed

Case #	Mutation	Gender	Thal A*β* phase	Braak NF stage	Age at onset	Age at death	*APOE* genotype	PMI (h)	Effects on A*β* production^a^
1	*APP* I716F	M	5	VI	31	36	33	15	A*β*1-40 ↓
									A*β*1-42↑
									A*β*1-42/A*β*1-40↑
2	*APP* A713T	M	5	VI	49	56	33	16	NA
3	*PSEN1* V89L	M	5	VI	48	57	23	9.5	NA
4	*PSEN1* E120G	M	5	VI	34	44	33	5.5	NA
5	*PSEN1* M139T	M	5	V	47	64	33	14.7	A*β*1-42/A*β*tot↑
6	*PSEN1* M139T	M	5	VI	48	57	33	15.2	–
7	*PSEN1* M139T	M	5	VI	45	53	33	5.3	–
8	*PSEN1* P264L	F	5	VI	45	56	44	6	A*β*1-40 = A*β*1-42↑
									A*β*1-42/A*β*1-40↑
9	*PSEN1* P264L	M	5	VI	53	60	34	7.2	–
10	*PSEN1* L286P	F	5	V	35	56	33	5	NA
11	*PSEN1* E280A	F	5	VI	47	54	33	5.5	A*β*1-40 = A*β*1-42↑
									A*β*1-42/A*β*1-40↑
12	*PSEN1* E280A	F	5	VI	42	50	33	7.5	–
13	*PSEN1* E280A	M	5	VI	44	52	33	4.8	–
14	*PSEN1* E280A	M	5	VI	47	56	33	3.3	–
15	*PSEN1* E280A	F	5	VI	49	62	33	4	–
16	*PSEN1* E280A	F	5	VI	37	47	33	2.3	–
17	*PSEN1* E280A	M	5	VI	49	55	44	2.8	–
18	*PSEN1* E280A	F	5	VI	50	60	33	2.8	–

*NA* not available, *NF* neurofibrillary, *M* male, *F* female

^a^Source: http://www.molgen.ua.ac.be/ADMutations

### Human CSF samples

A total of 60 CSF samples were included in this study. CSF samples from *PSEN1* mutation carriers were part of the Genetic Counseling Program (PICOGEN) [18] at the Hospital Clínic, Barcelona. This group included 10 subjects carrying *PSEN1* mutations (5 subjects with ADAD, global deterioration scale 3–5 and 5 presymptomatic mutation carriers), and 7 non-mutation carriers from the same family (Table 2). The clinical and CSF data of some of these patients have been previously reported [17]. Adjusted age was defined as the subject’s age relative to the median age of onset in the family. We also included 32 CSF samples from patients with dementia due to SAD and 11 age-matched healthy controls (mean age 74.6 ± 5.3 and 67.6 ± 4.0, respectively) obtained at the Hospital Sant Pau, Barcelona.

**Table 2:** Clinical and demographic data from *PSEN1* mutations carriers from whom CSF was analyzed

Group	*PSEN1* mutation	Age (years)	MMSE score	CSF A*β*42 levels (pg/ml)	Effects on A*β* production^a^
Healthy controls					
1	–	25.1	29	667	–
2	–	35.4	29	647	–
3	–	34.7	30	691	–
4	–	38.8	29	578	–
5	–	51.7	29	430	–
6	–	43.8	29	734	–
7	–	42.3	28	769	–
Presymptomatic *PSEN1* mutation carriers					
8	M139T	–	30	822	A*β*1-42/A*β*tot↑
9	M139T	–	30	753	–
10	M139T	–	28	655	–
11	M139T	–	29	505	–
12	K239N	–	29	1091	NA
Symptomatic *PSEN1* mutation carriers					
13	L235R	46	11	279	NA
14	L282R	46.3	22	199	NA
15	L286P	37.3	28	166	NA
16	L286P	42.6	24	163	NA
17	L286P	44.7	24	165	NA

The age has been omitted in presymptomatic mutation carriers to protect confidentiality

*MMSE* Mini-Mental State Examination, *NA* not available

^a^Source: http://www.molgen.ua.ac.be/ADMutations

### BACE-specific enzymatic activity assay

Tissues of human brain samples weighing 100–200 mg were homogenized with the proteoExtract™ Native Membrane Protein Extraction Kit (Calbiochem). BACE1 activity in human brain homogenates was measured as previously described [19, 20]. This BACE activity assay was based on an antibody capture assay in which activity was measured via fluorescent emission after the cleavage of a *β*-secretase substrate [19, 20]. To avoid the detection of other *β*-secretase activities, BACE was first captured via its C-terminal domain with anti-BACE1 antibody MAB5308 (mouse monoclonal anti-BACE, Chemicon) raised against BACE1 epitopes different from BACE2. Briefly, 96-well plates were coated overnight with the capture antibody MAB5308 at a dilution of 1:1,000 in 100 mM carbonate buffer at 4 °C. The plates were washed three times with phosphate-buffered saline (PBS, pH 7.0), and then blocked with a blocking reagent (25 % BlockAce; Dai-Nippon) for 6 h. The samples (50 μl of 0.004 wt/vol) were added to the wells containing 50 μl of Superblock^®^ blocking buffer (Pierce) in PBS and incubated for 1 h at 37 °C. The plates were washed 6 times with PBS, and the enzymatic reaction was carried out by incubation with 10 μM of the fluorogenic *β*-secretase substrate Arg-Glu(5-[aminoethyl] aminonaphthalene sulfonate [EDANS])-Glu-Val-Asn-Leu-Asp-Ala-Glu-Phe-Lys (4′-dimethylaminoazo-benzene-4-carboxylate[DABCYL])-Arg (Calbiochem) in acetate buffer at pH 4.1, which is optimal for BACE activity [19, 20]. Samples were incubated overnight at 37 °C, and the enzymatic reaction was measured using a Victor3 Wallac microplate reader (Perkin-Elmer).

BACE activity in human CSF was measured by incubating 10 μl of sample with 50 μl of BACE substrate (40 μM) overnight at 37 °C in acetate buffer with 100 mM sodium chloride (pH 4.1) containing 0.025 % BSA [60]. Fluorescence was measured at different time points with a Victor3 Wallac microplate reader with an excitation wavelength at 355 nm and emission wavelength at 486 nm. The concentration of BACE substrate used was that which best differentiated serial CSF dilutions over different time points. The enzymatic activity was calculated as ΔUF/min from the linear part of the reaction (between 2 and 24 h). The activity was completely inhibited by a BACE1 inhibitor verifying the specificity of the assay. The intra-assay and inter-assay coefficients of variation were 1.2 and 5.8 %, respectively.

### BACE1 protein, sAPP*α*, sAPP*β* and APP *β*-CTF assays

BACE1 protein, sAPP*α*, sAPP*β*, and APP *β*-CTF levels were measured in human brain samples or CSF using commercial kit assays (IBL). These assays are based on a solid-phase sandwich ELISA using specific anti-BACE or anti-APP antibodies. For sAPP*β* levels the cross-reactivity with human sAPP*β*-Sw and human sAPP*α* is 0.25 and 1.41 %, respectively, and for APP *β*-CTF levels the cross-reactivity with human sAPP*β* and human sAPP*α* is ≤0.1 %. BACE1 protein and APP *β*-CTF levels were measured in the membrane brain fraction while sAPP*α* and sAPP*β* levels were measured in CSF.

### Immunohistochemistry procedures

Detailed neuropathological studies were performed on multiple formalin-fixed, paraffin-embedded samples, as previously described [23]. For immunohistochemistry, dewaxed 5-μm-thick sections that included hippocampus, parahippocampal and temporo-occipital gyrus were immunostained in an automated stainer (DAKO Autostainer Plus) using the following antibodies: a rabbit anti-APP C-terminal (Sigma-Aldrich) recognizing the C-terminus (amino acids 676–695) of human APP 695, APP751 and APP770 at a dilution of 1:1,500; a mouse anti-APP N-terminal (Millipore, clone 22C11) antibody at a dilution of 1:50; and a mouse monoclonal anti-*β*A4-amyloid (DAKO, clone 6F/3D) antibody at a dilution of 1:400. To further evaluate the neuritic component of amyloid plaques, anti-ubiquitin (DAKO, polyclonal) and anti-hyperphosphorylated tau (Thermo Scientific, mc, clone AT8) antibodies were used at a dilution of 1:400 and 1:200, respectively. APP-immunoreactive structures were assessed semiquantitatively as follows: + mild (1–10 conglomerates of dystrophic neurites in one visual field using a 10× objective), ++ moderate (from 10 to 20 neuritic conglomerates), +++ abundant (>20 neuritic conglomerates), similar to the assessment of *β*-amyloid deposits [1]. For antigen retrieval, sections were immersed for 5 min in 98–100 % formic acid and heated for 20 min in a pressure cooking oven in 0.1 M sodium citrate buffer at pH 6.0. The reaction was visualized with 0.05 % diaminobenzidine and 0.01 % H_2_O_2_.

### Western blot

Human brain homogenates were electrophoresed in 5–16 % Tris-Tricine gels, transferred to 0.2 μm nitrocellulose membranes, and detected by immunoblotting with a rabbit anti-APP C-terminal (1:2,000; Sigma-Aldrich), a rabbit monoclonal N-terminus anti-BACE (D10E5, 1:1,000; Cell Signaling), a mouse monoclonal C-terminus anti-BACE (MAB5308, 1:1,000; Chemicon) or mouse monoclonal anti-tubulin (1:20,000; Sigma-Aldrich) antibodies. Specificity of the anti-BACE antibodies was verified by Western blot using brain homogenates from P7 BACE1−/− mice (a kind gift from Bart De Strooper [16]; Fig. S1). Incubation with primary antibodies was followed by detection with IR-fluorescent-conjugated antibody (LI-COR Biosciences). All blots were quantified by densitometric analysis and normalized to tubulin (Odyssey software, LI-COR Biosciences).

### *APOE* genotyping

*APOE* genotype was determined as previously described [23].

### Statistical analysis

Non-parametric statistical analysis (Kruskal–Wallis) was performed to analyze differences in BACE1, APP *β*-CTF, sAPP*β* protein levels and BACE activity. Correlation analysis between age and CSF BACE1 activity, APP *β*-CTF, and sAPP*β* protein levels was performed using the Spearman’s Rho test. Statistical significance for all the analyses was set at 5 % (*α* = 0.05). All data were analyzed using the Statistical Package for the Social Sciences (SPSS) version 20.0.

## Results

### BACE1 protein levels and activity are elevated in SAD but not in ADAD brains

**Fig. 1:**
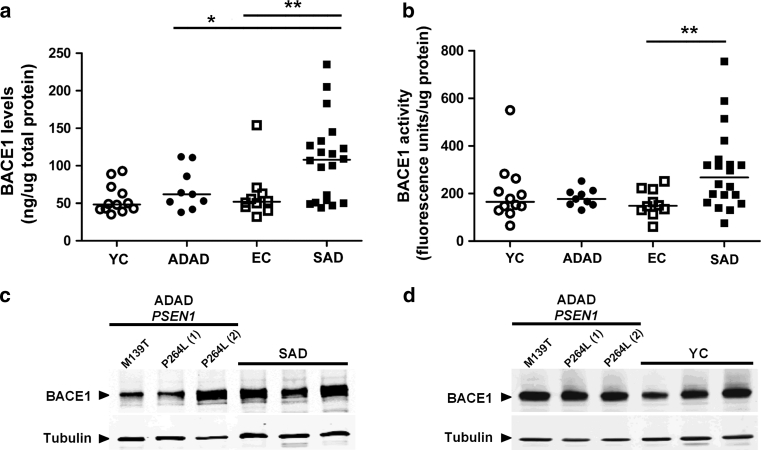
Brain BACE1 protein levels and activity in ADAD, SAD patients and controls. BACE1 protein levels and activity were measured in brain homogenates from ADAD and SAD patients, and from young (YC) and elderly (EC) controls. There was an increase in BACE1 protein levels (**a** **p = 0.01) and activity (**b** *p = 0.04) in the frontal cortex of SAD cases compared to age-matched elderly controls. No differences were detected between ADAD cases and age-matched controls in either brain BACE1 protein levels or activity (*p* = 0.91 and *p* = 0.42, respectively). There was a significant increase in BACE1 protein levels (**a** *p = 0.03) but not in BACE1 activity (**b**, n.s., *p* = 0.12) in SAD relative to ADAD cases. Western blot analyses using the BACE-specific antibody BC05 confirmed the increased in BACE expression in SAD compared to ADAD (**c**) and the lack of differences between ADAD and controls (**d**). Representative blots are shown

We first examined the initial proteolytic cleavage involved in A*β* generation, performed by BACE1 [37]. BACE1 protein levels and activity were measured in homogenates from the frontal cortex from ADAD cases, and compared with those from SAD cases and age-matched controls. Across the entire group (*n* = 51) BACE1 protein levels and activity positively correlated with age (*ρ* = 0.3, *p* = 0.03 and *ρ* = 0.28, *p* = 0.04, respectively). Consistent with other studies [19], there was no association of BACE1 protein levels or activity with PMI, gender or *APOE* genotype. BACE1 protein levels correlated with BACE1 activity in the entire sample (*ρ* = 0.29, *p* = 0.04). No differences were detected in either brain BACE1 protein levels or activity between ADAD cases and age-matched controls (Fig. 1a, b; *p* = 0.91 and *p* = 0.42, respectively). Consistent with previous reports [19, 27, 58], we found an increase in BACE1 protein levels (1.91-fold, Fig. 1a; *p* = 0.01) and activity (1.76-fold, Fig. 1b; *p* = 0.04) in the frontal cortex of SAD cases when compared to age-matched controls. There was a significant increase in BACE1 protein levels (*p* = 0.03) but not in BACE1 activity (*p* = 0.12) in SAD relative to ADAD cases. The levels of BACE1 protein in brain homogenates were also analyzed by Western blot using the specific anti-BACE1 antibody D10E5 (Fig. S1). These analyses confirmed the increase in BACE1 protein expression in SAD relative to controls and ADAD, as well as the lack of differences between ADAD and controls (Fig. 1c, d).

### CSF BACE1 expression and activity are not elevated in *PSEN1* mutation carriers or in SAD dementia cases

**Fig. 2:**
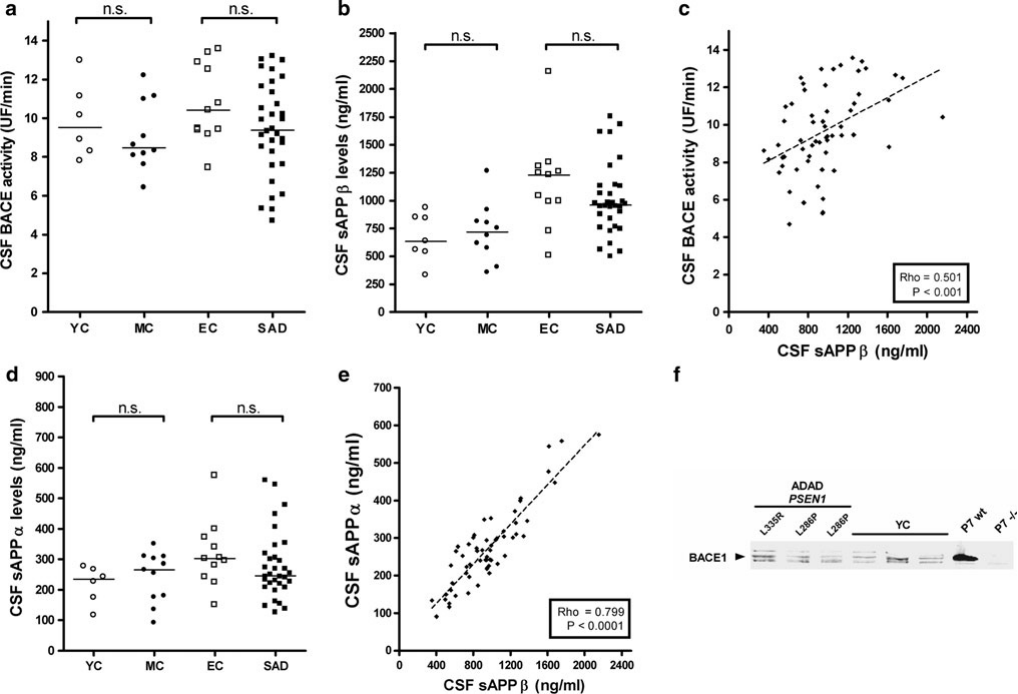
CSF BACE1 activity in *PSEN1* mutation carriers, SAD patients and controls. **a** CSF BACE1 activity was measured in non-mutation carriers controls (*YC*), *PSEN1* mutation carriers (*MC*), elderly controls (*EC*) and SAD patients. No differences were found between groups in CSF BACE1 activity. **b** CSF sAPP*β* levels were determined in YC, MC, EC and SAD patients. No differences were found between SAD cases and age-matched controls or between MC and YC. **c** CSF BACE1 activity and sAPP*β* levels showed a positive correlation in the entire subject sample (*ρ* = 0,501, *p* < 0.001). **d** CSF sAPP*α* levels were determined in YC, MC, EC and SAD patients. No differences were found between SAD cases and age-matched controls or between MC and YC. **e** CSF sAPP*β* and sAPP*α* levels showed a strong positive correlation in the entire subject sample (*ρ* = 0,799, *p* < 0.0001). **f** Western blot analyses of BACE1 protein levels using the specific anti-BACE1 antibody D10E5 showed no differences between *PSEN1* mutation carriers and non-carriers (*YC*). The specificity of the D10E5 was determined by using brain samples from 7-day-old (*P7*) wt and BACE 1−/− mice (Fig. S1). A representative blot is shown

We next tested whether an increase in BACE1 expression or activity was present in ADAD in the CSF. We obtained CSF samples from a cohort of subjects recruited from a genetic counseling program for familial dementias [17, 18]. We used a fluorogenic CSF BACE1 enzymatic activity assay to measure BACE1 activity in CSF samples from 17 *PSEN1* mutation carriers and non-carriers (Table 2), subjects with SAD dementia (*n* = 32) and healthy controls (*n* = 11). CSF BACE1 activity did not correlate with age, MMSE score or CSF A*β*42 levels in the entire sample (*n* = 60) or with the adjusted age in *PSEN1* mutation carriers (*n* = 10). When analyzed according to clinical or mutation status, no differences in CSF BACE1 activity were detected among *PSEN1* mutation carriers and non-mutation carriers (*p* = 0.85) or between SAD cases and controls (*p* = 0.1; Fig. 2a). As an additional measure of APP processing, we analyzed CSF levels of sAPP*β*, a soluble fragment generated by BACE cleavage. Levels of sAPP*β* correlated positively with age ( *ρ* = 0.279; *p* = 0.03) but not with MMSE score or CSF A*β*42 levels in the entire sample. We found no differences in CSF sAPP*β* levels between *PSEN1* mutation carriers and non-mutation carriers (*p* = 0.85; Fig. 2b) or between SAD dementia cases and age-matched controls (*p* = 0.12; Fig. 2b). CSF BACE1 activity and sAPP*β* levels showed a positive correlation in the entire subject sample (*ρ* = 0.501, *p* < 0.001; Fig. 2c). Almost identical results were found when CSF sAPP*α* levels were measured (Fig. 2d). This is not surprising since CSF sAPP*β* and sAPP*α* levels showed a strong positive correlation in the entire subject sample (*ρ* = 0.799, *p* < 0.0001; Fig. 2e), as in previously reported studies [33, 59]. To examine whether there was any difference in CSF BACE1 protein levels, Western blotting was carried out using the specific anti-BACE1 antibody D10E5 and no differences were detected between *PSEN1* mutation carriers and non-mutation carriers (Fig. 2f). Overall, no changes in CSF BACE1 activity or expression could be detected in subjects with *PSEN1* mutations or SAD compared to age-matched controls.

### Brain APP *β*-CTF levels are higher in ADAD than SAD

**Fig. 3:**
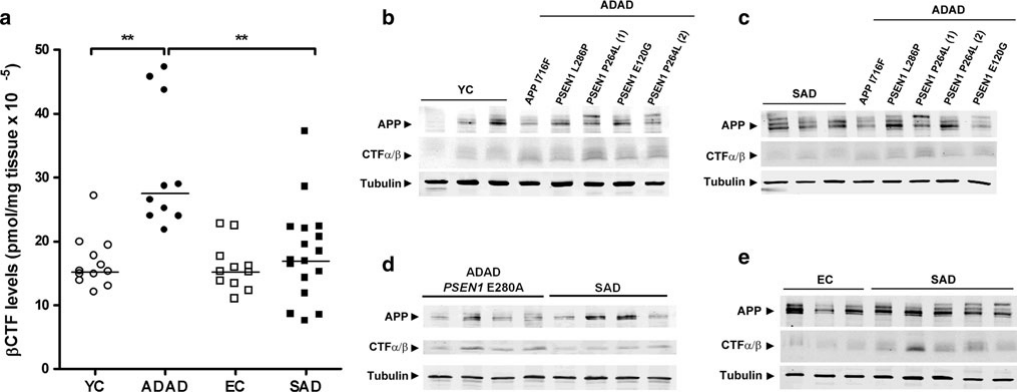
Brain APP *β*-CTF levels are elevated in ADAD. APP *β*-CTF levels were measured in membrane fractions in brain homogenates from ADAD, SAD and controls (**a**). ADAD cases showed higher APP *β*-CTF levels than age-matched controls and SAD (**p < 0.01). These differences were confirmed by Western blot in samples from patients with ADAD, SAD and controls. APP CTF accumulation was observed in ADAD cases compared to age-matched controls (**b**) and to SAD cases (**c**, **d**). APP CTF accumulation was also observed by Western blot in SAD cases compared to controls despite the fact that it did not reach statistical significance in the ELISA assay (**e**)

Using ELISA, we then determined the levels of APP *β*-CTF, a protein fragment generated from full-length APP in frontal cortex brain homogenates obtained from patients with ADAD, SAD and controls. The APP *β*-CTF fragment is generated by BACE and processed by *γ*-secretase to release A*β* peptides [37]. We found that ADAD cases showed a prominent APP *β*-CTF accumulation when compared to age-matched controls and to SAD cases (both *p* < 0.001, Fig. 3a). SAD cases did not show statistically significant higher APP *β*-CTF levels than age-matched controls by ELISA (*p* = 0.47, Fig. 3a), despite being elevated by Western blot (Fig. S2). No differences were found between young and elderly controls. The levels of APP *β*-CTF were not influenced by age, gender or *APOE* genotype. The results of the ELISA were confirmed by Western blot analysis in our subject sample and in patients with the E280A *PSEN1* mutation (Fig. 3b–d; Fig. S2). Among ADAD cases, the *APP* A713T and some *PSEN1* mutations (P264L, P286P) displayed higher levels of APP C-terminal fragments than others (M139T, V89L).

### APP-immunoreactive dystrophic neurites and aggravated neuritic component in ADAD

**Fig. 4:**
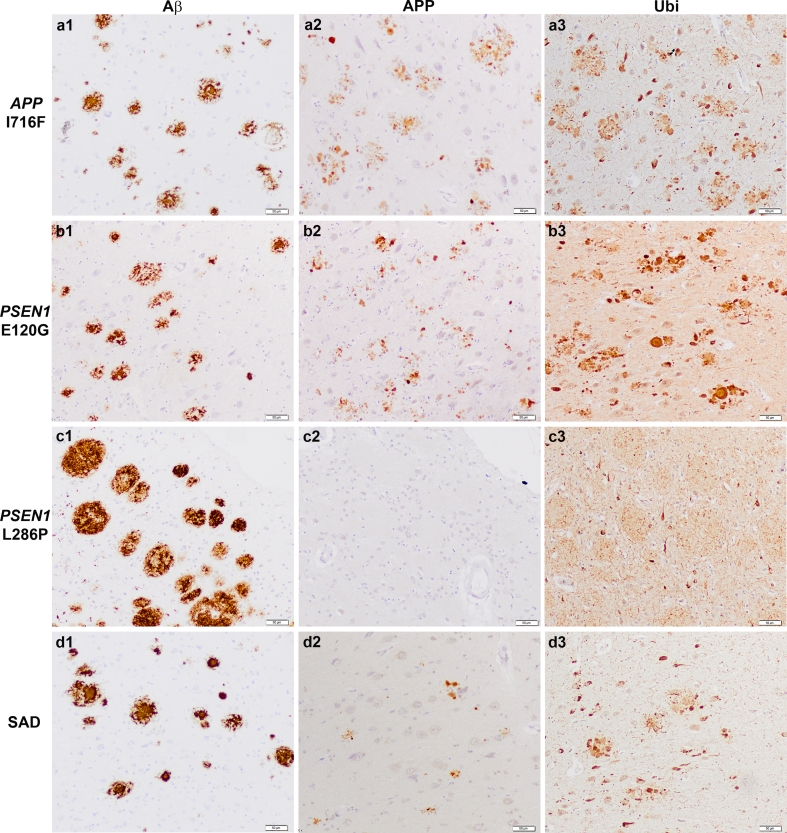
APP accumulates in dystrophic neurites in ADAD. Immunohistochemistry for A*β*, APP and ubiquitin on representative brain sections from ADAD subjects carrying the *APP* I716F (**a1**–**a3**), the *PSEN1* E120G (**b1**–**b3**), *PSEN1* L286P (**c1**–**c3**) mutations and from one patient with SAD (**d1**–**d3**). Note frequent A*β* deposits, APP- and ubiquitin-positive bulbous dystrophic neurites in subjects with *APP* I716F and *PSEN1* E120G mutations in contrast to the nearly lack of APP-positive neurites in subject with the *PSEN1* L286P mutation where cotton-wool plaques predominate. In the latter case, ubiquitin immunostains delicate intermingled neurites (**c3**). In the SAD case, abundant mature A*β* deposits (**d1**) contrast with the few APP-(**d2**) and prominent ubiquitin-immunoreactive dystrophic neurites. *Bar* 50 µm

Since our biochemical data indicated elevated APP *β*-CTF levels in ADAD compared to SAD and controls, we next evaluated the distribution of APP accumulation. We performed immunohistochemical studies on brain sections from ADAD cases using either an anti-APP C-terminal or an anti-APP N-terminal antibody. As previously described [13, 30, 43], we confirmed that antibodies rose against APP labeled dystrophic neurites of senile plaques in SAD using both, anti-C-terminal and anti-N-terminal antibodies. Both APP antibodies also detected APP epitopes in dystrophic neurites of senile plaques in ADAD (Fig. S3). Semiquantitative assessment of the neuritic component associated to amyloid plaques in parahippocampal and temporo-occipital cortices revealed that patients with ADAD had a more prominent neuritic component than those with SAD (Fig. 4; Table 3) independently of the number of A*β* plaques. This was also observed using anti-ubiquitin (Fig. 4) and anti-phosphorylated tau antibodies (Fig. S3 and data not shown). Cases with predominant cotton-wool plaques (*PSEN1* P264L, *PSEN1* L286P) showed fewer APP-immunoreactive dystrophic neurites than ADAD cases with neuritic plaques (Fig. 4).

**Table 3:** Relationship between dystrophic neurites and A*β* deposits in ADAD cases

Case	Mutation	APP-immunoreactive dystrophic neurites	A*β* deposits
1	*APP* I716F	+++	Abundant mature and primitive plaques
2	*APP* A713T	+++	Abundant mature and primitive plaques. Few diffuse plaques
3	*PSEN1* V89L	+++	Abundant mature and primitive plaques
4	*PSEN1* E120G	+++	Abundant mature and primitive plaques
5	*PSEN1* M139T	++	More primitive plaques than mature plaques
6	*PSEN1* M139T	++	Abundant mature and primitive plaques
7	*PSEN1* M139T	+++	Abundant mature and primitive plaques
8	*PSEN1* P264L	+	Predominantly cotton-wool plaques
9	*PSEN1* P264L	++	Abundant cotton-wool plaques mixed with mature and primitive plaques
10	*PSEN1* L286P	+	Predominantly cotton-wool plaques

## Discussion

The main finding in the current study is that ADAD and SAD display distinct profiles in BACE protein and activity, and in APP *β*-CTF levels in the brain. While no apparent increase in brain BACE1 protein levels or activity was observed in ADAD, both were clearly elevated in SAD. Accumulation of APP *β*-CTF was higher in the brain in ADAD than in SAD and controls. No changes in BACE measures were observed in the CSF between ADAD and SAD.

Research in ADAD has been instrumental as a model to understand the pathogenesis of SAD and to guide the current development of anti-amyloid strategies. The mainstream paradigm claims that ADAD and SAD share similar clinical and pathological phenotypes as well as common mechanisms of disease, irrespective of the initiation factors [6, 54]. However, very few studies have investigated the differences in APP processing between ADAD and SAD in the human CNS to support this view. Previous studies have mainly focused on A*β* isoforms, tau or p-tau in the CSF [5, 44, 46, 49] or A*β* isoforms in human brain [45, 49]. The findings suggest a specific CSF profile of A*β* isoforms in ADAD, with low levels of A*β*1-37, A*β*1-38, A*β*1-39 and A*β*1-42 compared with SAD [5, 44]. Whether other relevant aspects of APP processing differ between ADAD and SAD remains unknown.

BACE1 is a type-I transmembrane protease highly expressed in neurons [11]. Previous studies have demonstrated that BACE1 protein levels and activity are elevated approximately twofold in the brain of SAD patients [9, 19, 27, 58], suggesting that this feature might initiate or contribute to brain A*β* accumulation [34]. Our data confirmed the increased BACE1 levels and activity in SAD brains, but no increase could be detected in ADAD cases compared to age-matched controls. Although the increase in brain BACE1 protein levels in SAD relative to ADAD cases might reflect a difference in chronological age between groups, the increment in brain BACE1 activity and protein levels in SAD compared to age-matched controls suggests a disease-specific effect. Our data differ from the only previous study that had examined BACE1 expression or activity in ADAD brains [22], where the authors reported an increase in BACE1 mRNA levels and activity in 11 ADAD cases carrying 10 different *PSEN1* mutations. The strengths of the current study are the use of age-matched controls without any brain lesion, the selection of a region in which elevated BACE1 activity had been previously detected in SAD [34], and the investigation of CSF samples obtained from *PSEN1* mutation carriers. Our findings lend support to other studies that reported no change in BACE1 expression or activity in either APP or PS1 mutant-transfected cells or APP×PS1-transgenic mice [4, 20, 34].

In contrast with our findings in human brain, we did not find any differences in CSF BACE activity or expression, or in sAPP*β* levels between groups. The lack of increase in CSF BACE1 activity or sAPP*β* levels in cases with SAD dementia compared to healthy controls is in agreement with most recent studies [48, 59]. The present data together with previous work [48, 59, 60] suggest that BACE1 activity may become elevated at the stage of mild cognitive impairment, and then decrease over time as disease progresses [48]. Our data obtained in patients with SAD dementia also indicate that CSF BACE1 activity does not parallel brain BACE1 activity, at least in the advanced stage of the disease. While BACE1 activity and protein levels in the brain tend to increase in late-stage AD, BACE1 activity in the CSF would stabilize or even decrease, perhaps as a result of a reduction in global neuronal function [48]. More generally, this observation indicates that CSF may not accurately reflect the changes in the local intracellular or extracellular environment [10, 57]. Taken together, our results indicate that brain BACE1 up-regulation is characteristic of SAD, but is not a salient feature in many ADAD-associated mutations. Previous studies [26, 27, 47] have shown that increased BACE1 expression in SAD is due to post-translational regulation mechanisms and that BACE1 mRNA levels are unchanged. One possible explanation is that the increase in BACE1 in SAD results from the interaction of Alzheimer pathology with diverse factors associated to aging, such as oxidative stress, inflammatory changes or microRNA dysregulation, conditions known to increase BACE1 expression and activity in cell culture [12, 26, 50, 55].

The lack of increase in BACE1 in ADAD has clinical implications as BACE1 has become an attractive drug target for AD intervention [11]. Although inhibitor development has proved to be highly challenging, some promising BACE1 inhibitors as well as other strategies, such as immunization with anti-BACE1 antibodies, have been developed [11]. The lack of increase in brain or CSF BACE1 expression or activity in ADAD in our study suggests that BACE1 is a less attractive target for families with ADAD than for patients with SAD. Nonetheless, it is still possible that BACE1 inhibition may prove to be effective as a preventive therapy in subjects with *APP* or *PSEN1* mutations. This is a relevant and timely topic since clinical trials in ADAD are imminent.

Another important finding derived from our study is the higher accumulation of APP *β*-CTFs in the brain of ADAD cases than in SAD patients and controls. The APP *β*-CTF fragment is generated by BACE and processed by *γ*-secretase to release A*β* peptides [37]. The main explanation for the APP *β*-CTF accumulation in SAD has been the overproduction due to increased BACE1 protein levels and activity [58]. However, the lack of elevated BACE1 in ADAD points to other underlying mechanisms. It has been suggested that *PSEN* mutations alter the conformation of the *γ*-secretase complex [7, 14]. This change could be a plausible mechanism by which *PSEN* mutations lead to *γ*-secretase dysfunction and the formation of longer A*β* peptides in ADAD [7, 15]. Since APP *β*-CTF are processed by *γ*-secretase, it is possible that elevated APP *β*-CTF may be the result of a dysfunctional *γ*-secretase. A recent study has shown that ADAD-associated mutations do not consistently affect kinetic activity [15] excluding the possibility that mutations inhibit *γ*-secretase. However, conformational changes in *γ*-secretase may subtly slow substrate processivity which could increase *β*-CTF in ADAD. Other possible mechanisms underlying APP *β*-CTF accumulation in ADAD include impaired macroautophagy as it has been shown that APP *β*-CTF is rapidly cleared by autophagy under physiological conditions [56]. In any case, the accumulation of APP CTFs has been shown to be neurotoxic by itself [29, 31, 39, 42]. This phenomenon has also been observed in wild-type mice or transgenic APP mouse models after treatment with classical *γ*-secretase inhibitors [8, 40] or after inactivation of *PSEN1* [51]. In both these situations, APP CTFs accumulate at the presynaptic terminals, likely impairing synaptic plasticity and long-term memory [40, 51]. Interestingly, APP CTF accumulation has been postulated, together with inhibition of Notch processing [15], as a possible mechanism underlying cognitive side effects in patients with AD treated with the *γ*-secretase inhibitor semagacestat [8, 37]. Although the precise cause of APP CTF accumulation in ADAD deserves further investigation, it is likely that this feature acts as an active component of the disease that may contribute to the metabolic and cytoskeletal derangement and neurodegeneration.

Our findings also demonstrate that the neuritic component is more prominent in ADAD cases than in SAD. It has previously been shown that full-length APP accumulates in dystrophic neurites in SAD [13, 21, 28, 30, 43] and that this accumulation is an early event that occurs prior to tau accumulation [13]. Our results extend these findings to ADAD and show a more severe neuritic component in ADAD than in SAD. The wide variety of neuronal proteins found in AD in dystrophic neurites has been increasingly recognized as a failure of the autophagic-lysosomal pathway [41]. In addition, it has been shown that PS1 is essential for lysosomal proteolysis and autophagy and that PS1-null or PS-ADAD fibroblasts display marked autophagy impairment [32, 41]. This defect could account for our observation of numerous and enlarged dystrophic neurites in ADAD as compared to SAD. Finally, the contribution of APP-immunoreactive dystrophic neurites to parenchymal amyloid deposition seems unlikely because at least one half of diffuse plaques, which may represent the earliest stage of the amyloid plaque, do not contain APP-immunoreactive neuritic profiles [13, 30] and we did not observe APP epitopes in dystrophic neurites in ADAD cases with cotton-wool plaques.

The main limitation in the present study is that as only the frontal cortex region was analyzed, we cannot exclude the possibility that other brain areas might have shown different results. Besides, our study only included cases carrying either two *APP* mutations or nine *PSEN* mutations, and whether our findings are generalizable to all ADAD cases requires further investigation. Finally, it is worth mentioning that the two *APP* mutations investigated herein are close to the *γ*-secretase cleavage site, and they were predicted to affect *γ*-secretase processing on a similar way to *PSEN* mutations. It is possible that other *APP* mutations located outside of the *γ*-secretase cleavage site may have shown different effects.

In summary, the data presented herein reinforce the different physiopathological mechanisms underlying the A*β* production/clearance imbalance in SAD and ADAD. These differences in APP processing may contribute to explain the lack of alignment between studies in humans and in AD animal models. A deeper understanding of the common and divergent fundamental pathogenic mechanisms in ADAD and SAD is needed to fine-tune and accelerate drug development in AD.

## Electronic supplementary material

Below is the link to the electronic supplementary material.

Supplementary material 1 (TIFF 374 kb) Fig. S1. Specificity of the anti-BACE antibodies used in this study. The specificity of two anti-BACE antibodies (D10E5, N-terminus, Cell Signaling; MAB5308, C-terminus, Chemicon) was tested using brain homogenates from a SAD case, 7-days old (P7) wild type and BACE1 -/- mice (a kind gift from Bart De Strooper [16]). Western blot analysis showed absence of BACE1 immunoreactivity (arrowhead) in samples from P7 BACE1 -/- mice.

Supplementary material 2 (TIFF 182 kb) Fig. S2. Western blot analyses of APP CTFs in human brain samples. APP-FL and APP CTFs were detected by using a rabbit APP C-terminal antibody. Cell lysates from CHO cells overexpressing APP treated with the *γ*-secretase inhibitor DAPT were used as a control (**a**). Densitometric analysis of the ratio APP CTF*α*-*β*/APP-FL from YC and ADAD (**b**), SAD and ADAD (**c**), and EC and SAD (**d**). Values represent the mean of al least three indendent experiments. Values are expressed as a % of controls (**b, d**) or SAD (**c**).

Supplementary material 3 (TIFF 9238 kb) Fig. S3. Anti-APP antibodies label dystrophic neurites of senile plaques in ADAD. Both anti-C-terminal (**a1**) and anti-N-terminal (**a2**) APP antibodies detect dystrophic neurites in a patient with the *APP* I716F mutation. In addition to APP, tau (**a3**) and ubiquitin (**a4**) antibodies label the neuritic component of amyloid plaques. *Bar* 50 µm
