# Supplementary figures and images for: Distinct patterns of APP processing in the CNS in autosomal-dominant and sporadic Alzheimer disease
Source: Acta Neuropathol. 2012 Dec 6;125(2):201–13. doi: 10.1007/s00401-012-1062-9 (PMC3623032; doi:10.1007/s00401-012-1062-9)

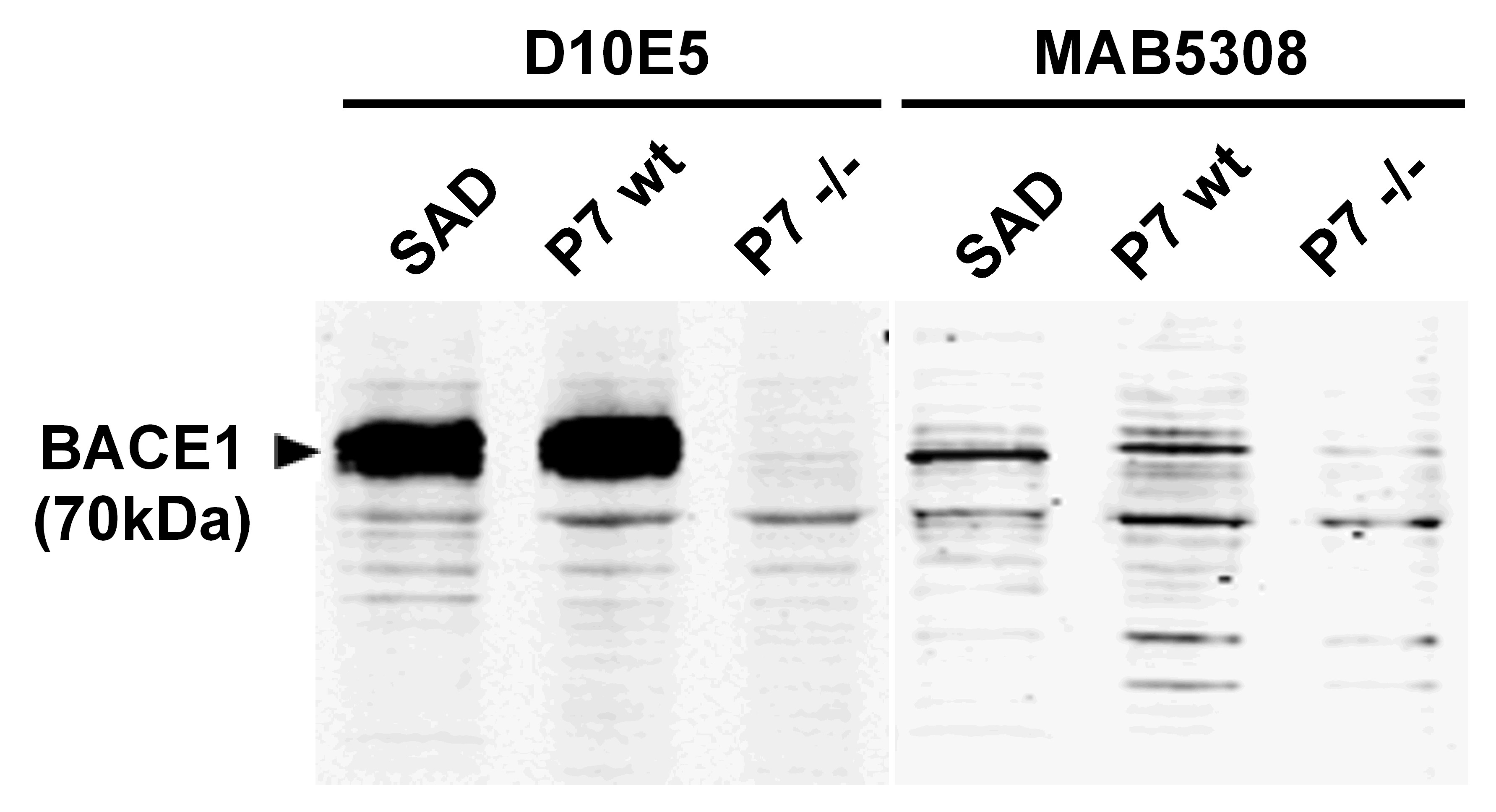

Supplement: Supplementary file 1 — Fig. S1. Specificity of the anti-BACE antibodies used in this study. The specificity of two anti-BACE antibodies (D10E5, N-terminus, Cell Signaling; MAB5308, C-terminus, Chemicon) was tested using brain homogenates from a SAD case, 7-days old (P7) wild type and BACE1 -/- mice (a kind gift from Bart De Strooper [16]). Western blot analysis showed absence of BACE1 immunoreactivity (arrowhead) in samples from P7 BACE1 -/- mice. [file 401_2012_1062_MOESM1_ESM.jpg]

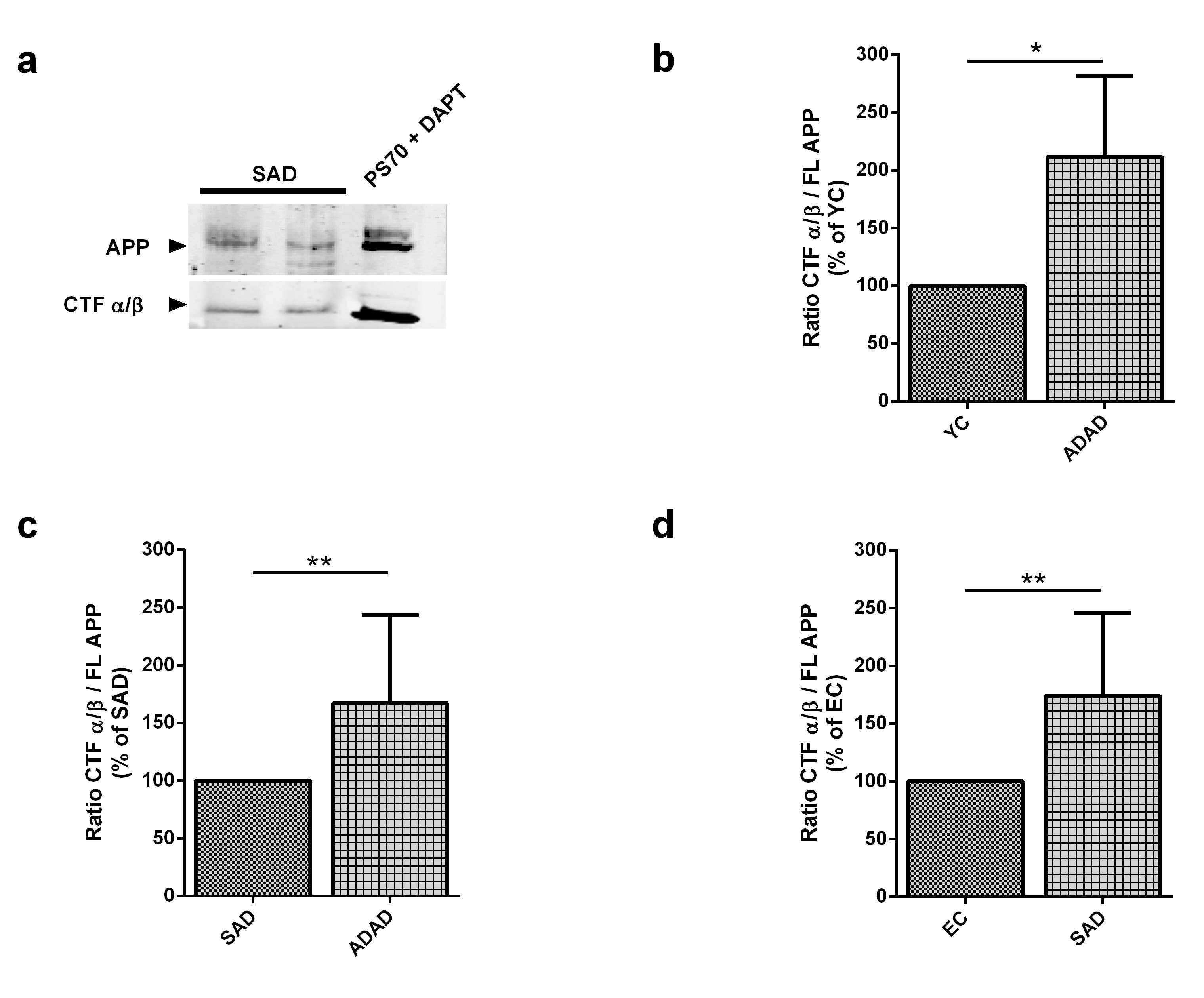

Supplement: Supplementary file 2 — Fig. S2. Western blot analyses of APP CTFs in human brain samples. APP-FL and APP CTFs were detected by using a rabbit APP C-terminal antibody. Cell lysates from CHO cells overexpressing APP treated with the γ-secretase inhibitor DAPT were used as a control (a). Densitometric analysis of the ratio APP CTFα-β/APP-FL from YC and ADAD (b), SAD and ADAD (c), and EC and SAD (d). Values represent the mean of al least three indendent experiments. Values are expressed as a % of controls (b, d) or SAD (c). [file 401_2012_1062_MOESM2_ESM.jpg]

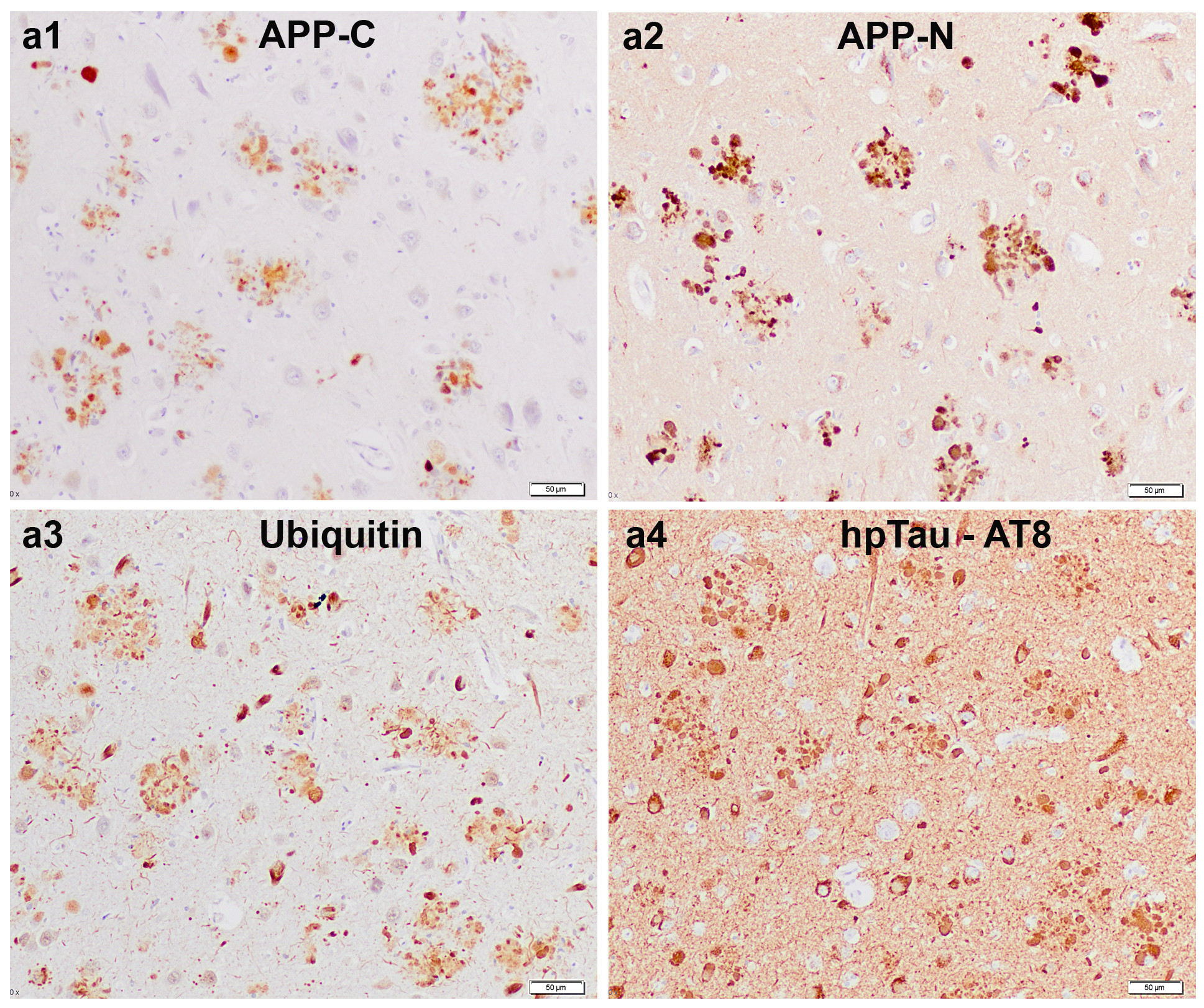

Supplement: Supplementary file 3 — Fig. S3. Anti-APP antibodies label dystrophic neurites of senile plaques in ADAD. Both anti-C-terminal (a1) and anti-N-terminal (a2) APP antibodies detect dystrophic neurites in a patient with the APP I716F mutation. In addition to APP, tau (a3) and ubiquitin (a4) antibodies label the neuritic component of amyloid plaques. Bar 50 µm [file 401_2012_1062_MOESM3_ESM.jpg]
